# The Blood Virome: A new frontier in biomedical science

**DOI:** 10.1016/j.biopha.2024.116608

**Published:** 2024-05-03

**Authors:** Abraham J. Kandathil, David L. Thomas

**Affiliations:** Department of Medicine, Johns Hopkins University School of Medicine, Baltimore, MD, USA

**Keywords:** Blood, Virome, Human health, Eukaryotic viruses, Commensal, Metagenomic sequencing

## Abstract

Recent advances in metagenomic testing opened a new window into the mammalian blood virome. Comprised of well-known viruses like human immunodeficiency virus, hepatitis C virus, and hepatitis B virus, the virome also includes many other eukaryotic viruses and phages whose medical significance, lifecycle, epidemiology, and impact on human health are less well known and thus regarded as commensals. This review synthesizes available information for the so-called commensal virome members that circulate in the blood of humans considering their restriction to and interaction with the human host, their natural history, and their impact on human health and physiology.

## 1. Introduction

The virome is quite possibly the most expansive component of the microbiome with an estimated 10^31^ members. [1] The virome includes viruses that infect host cells, bacteria, archaea, and fungi, as well as the fossilized viral remains archived in host chromosomes. [2] In mammals, there may be 10-fold more viruses than bacteria. [3] Although most medical research has focused on viruses with established impact on human health, [4,5] recent advances in sequencing have revealed novel viruses, viruses in novel host tissues, and the poorly classified ‘biological dark matter’. [5]

Viruses are found in all tissues; a subset is persistently detected in blood (Table 1) [5–8]. For some like HIV, hepatitis C virus (HCV), and hepatitis B virus (HBV), the medical importance and virology are well known. However, for others no medical significance has been discovered, and they are accordingly often referred to as commensals. Though we also adopt that language, whether these viruses have any medical or physiological significance remains largely uninvestigated. These commensal blood-borne human viruses are the focus of this review. Eukaryotic viruses with incomplete nucleotide sequences, viral sequences from non-eukaryotic hosts, transcripts of viral sequences integrated in human chromosomes, and rare instances of viral sequences detected in human blood with no additional information were considered outside the scope of this review and are not discussed in detail.

## 2. History of blood-born commensal viruses

The blood virome has been progressively revealed by advances in molecular virology. In the 1990s representative differential analysis (RDA) and polymerase chain reaction (PCR) were used to discover human pegivirus. [9] Initially, two new flaviviruses were detected by RDA in the plasma of a tamarin (*Sanguinus sp)*. [10] By broadening (degenerating) PCR primers based on the helicase sequences of the tamarin viruses, the investigators discovered pegivirus in humans. [9] The initial discovery of human pegivirus in the blood of persons with post-transfusion hepatitis led to the mistaken assumption the virus caused hepatitis and naming of the virus as hepatitis G. [11] Subsequently, the new virus was also recognized in the blood of humans without hepatitis, and those with the virus in blood were no more likely to have hepatitis than those without; the name GB virus-C or human pegivirus (HPgV) was sustained. [12,13] The Stapleton lab detected interactions between HPgV and HIV. [14] However, no other impact on human health has been confirmed.

In 1997, RDA was used to identify a novel viral DNA sequence named as TT virus in the serum of an individual (TT) with posttransfusion hepatitis. [15] As with HPgV, that association with clinical hepatitis is now recognized as coincidental; to date, no disease association has been confirmed (see sections 3.4 and 4.4).

In the past decade, virus discovery was advanced using metagenomic next generation sequencing (mNGS) approaches. Unbiased mNGS may reveal unexpected viral elements such as the diversity of anelloviruses, discovery of human hepegivirus (HHPgV), and ekpoma viruses. [7,8,16, 17] Despite these advances, as much as 95% of sequence reads remain unclassified suggesting more discoveries will come. [5]

## 3. DNA commensals in human blood virome

Globally, more than half the world’s healthy population have a circulating DNA virome which includes anelloviruses, genomoviruses, and phages. [5,18,19] Members of family *Anelloviridae* are genetically diverse, 30–32 nm, non-enveloped virions with negative-sense, single-stranded circular genomes. [20] Although there are at least 30 genera described in animals, humans are infected chiefly by three genera–*Alphatorquevirus* [*Alphatorquevirus homin* species (TTV)], *Betatorquevirus* [*Betatorquevirus homin* species (TTMV)], and *Gammatorquevirus* [*Gammatorquevirus homidi* species (TTMDV)]. [20] Each genus has multiple species, and co-infection with multiple species is routinely observed in humans. [17]

The family *Genomoviridae* includes 20–22 nm, non-enveloped virions with single-stranded circular genomes. [21] However, unlike anelloviruses, the ambisense genomovirus genome encodes rolling-circle replication initiation protein (Rep) and a capsid protein. [21] Hence, they are grouped along with other circular, Rep-encoding single-stranded (CRESS) DNA viruses. [22] The *Genomoviridae* family is composed of 10 genera with sequences identified in human blood belonging to genera *Gemykibivirus* and *Gemyvongvirus*. [21] In human blood, sequences have been observed in persons living with HIV and blood donors. [23,24] Genomovirus sequences have also been observed in a variety of environmental, animal, and plant specimens. [25]

Although the infecting cell is unclear, phages belonging to family *Myoviridae*, *Siphoviridae*, *Podoviridae*, and *Microviridae* have been reported in human blood. [5] However, many others are likely missed since there is little sequence homology between contigs identified in blood and the species used to build matching databases. [5]

While transmission via blood might have contributed to initial spread of human endogenous retroviruses (HERV) infection among humans, infectious virions have not been reported in blood. However, viral transcripts of integrated HERV have been detected in blood of healthy individuals. [26]

### 3.1. Epidemiology

Anelloviruses have been detected in blood within the first month of life, and appear to accumulate and vacillate during that year. [27] One study detected more *beta*- and *gammatorque* genera in infants, unlike adults in whom *alphatorqueviruses* are dominant. [27] There are parallels between the timing of first anellovirus detection in blood and stool but no direct evidence that virus in blood originates from replication in gut. [27,28] Anelloviruses are detected in breast milk, but it is not clear if infants acquire anelloviruses from breastfeeding. [27]

Anellovirus prevalence appears to increase with age. Focosi et al. observed an increase in TTV abundance among 1000 healthy Italian blood donors with age over a span of 50 years. [29] Accumulation of TTV suggests that the rate of acquisition and persistence exceeds virus clearance. Only a few studies have tested that hypothesis. In one longitudinal study, blood samples were studied in 78 people who inject drugs (PWID). [30] Among 56 in whom TTV were initially detected, 43 were also positive a mean of 1.86 years later. Similarly, persistence of *Alphatorquevirus homin7* was found in one of seven over a mean of 29 months. [30]

Among adults, there is direct evidence of blood borne transmission. [7,17] Anellovirus abundance increases after transfusion.[7] Interestingly, for reasons that are not clear, some donor anellovirus lineages are newly detected (and persistent) while other donor lineages are not detectable in recipients. [17] The volume of a blood donation is sufficiently large that factors other than exposure and inoculum size appear to determine the membership of each person’s anellome. There is also evidence of blood transmission from studies of PWID who have higher anellovirus prevalence and richness compared to controls who reported no injection drug use. [30] One study also observed that accumulation of anelloviruses in blood might be a harbinger for the risk of HCV among PWID. [30] Blood borne transmission has also been documented in nonhuman primates studies. [31,32]

Thus, anelloviruses are acquired by humans in infancy, most plausibly by ingestion, and are acquired by adults by percutaneous exposures. Whether infants may acquire infection in utero or at the time of delivery and whether adults continue to acquire anelloviruses by ingestion or other exposures like sexual intercourse or household/fomite contact remain unknown.

The epidemiology of human genomovirus infection is only beginning to unfold. There are at least 6 species found in humans. [21] *Gemykibivirus humas1* was first identified in the serum of an individual with multiple sclerosis, but is not linked to the disease. [33] Another species *Gemykibivirus humas2* was recently identified in Brazilian blood donations in which blood borne viral pathogens were identified. [34] A separate study of 450 healthy blood donors from multiple geographical locations within Brazil also detected *Gemykibivirus humas2* species DNA in 7.8%. [18] Regional differences in prevalence were noted with the highest prevalence (15.3%) from the Amazon and lowest (2.0%) from South Brazil. [18] In contrast, 128 HIV-positive and 256 HIV- negative plasma samples in France did not reveal any gemykibivirus viremia. [23] Gemykibivirus reads have also been detected in other metagenomic studies from cervico-vaginal and fecal sources. [35,36] But, the source of viremia and basis for differences in prevalence remain unknown.

### 3.2. Non-human mammalian analogs

Anellovirus sequences have been observed in a wide range of mammals including primates, rodents, and bats. [20] Studies done using blood indicate a high prevalence among healthy, non-human mammals. [37,38] Based on phylogenetic analysis sequences observed in primates, these isolates were assigned to *alpha-*, and *betatorqueviruses* genera. [37] One instance of cross species TTV infection between humans and chimpanzee has also been reported. [32]

Though the host range of genomovirus is largely unknown the majority of full-length viral sequences have been recovered from non-human specimens. [21]

### 3.3. Microbiology

Given the absence of culture models, most of what is known about the replication of anelloviruses including the host cell is inferred from *in vivo* detection of replication intermediates. [39,40] For example, double stranded TTV DNA has been reported in bone marrow and liver, and in liver, negative and positive strand DNA have been found. [39,40] One study reported detection of TTV mRNA splice variants - 2.8 kb, 1.2 kb, and 1 kb in the bone marrow of a patient with acute myeloblastic leukemia. [41] TTV mRNA has also been identified in ovaries, blood, and heart during data mining of RNAseq data generated by the Genotype-Tissue Expression (GTEx) project. [42]

In pediatric allogenic HSCT recipients, TTV DNA was observed in granulocytes (CD15+) from blood (0.1–50 copies/cell) and bone marrow (1–63 copies/cell) of all participants. However, only 10% of immunocompetent children had TTV in granulocytes (0.01–8 copies/cell). Further indirect evidence for anellovirus infection of circulating blood cells is inferred from the higher viremia observed in whole blood compared to plasma and serum fractions. [29] However, since anelloviruses circulate in blood, it is difficult to prove that detection even of replicative intermediates in a tissue proves replication occurs in those cells. It is also conceivable that more than one cell type supports infection.

Further work also needs to be done to understand the steps involved in replication. A recent structure based study indicated that anelloviruses likely replicate using different viral/host factors compared to Cressdnaviricota. [22,43] The extremely high diversity of anellovirus genomes is unusual for DNA viruses and largely thought to reflect recombination occurring across the entire genome. [17] Cocirculation of multiple anellovirus lineages supports that hypothesis. [17]

### 3.4. Medical significance

**Fig. 1.**
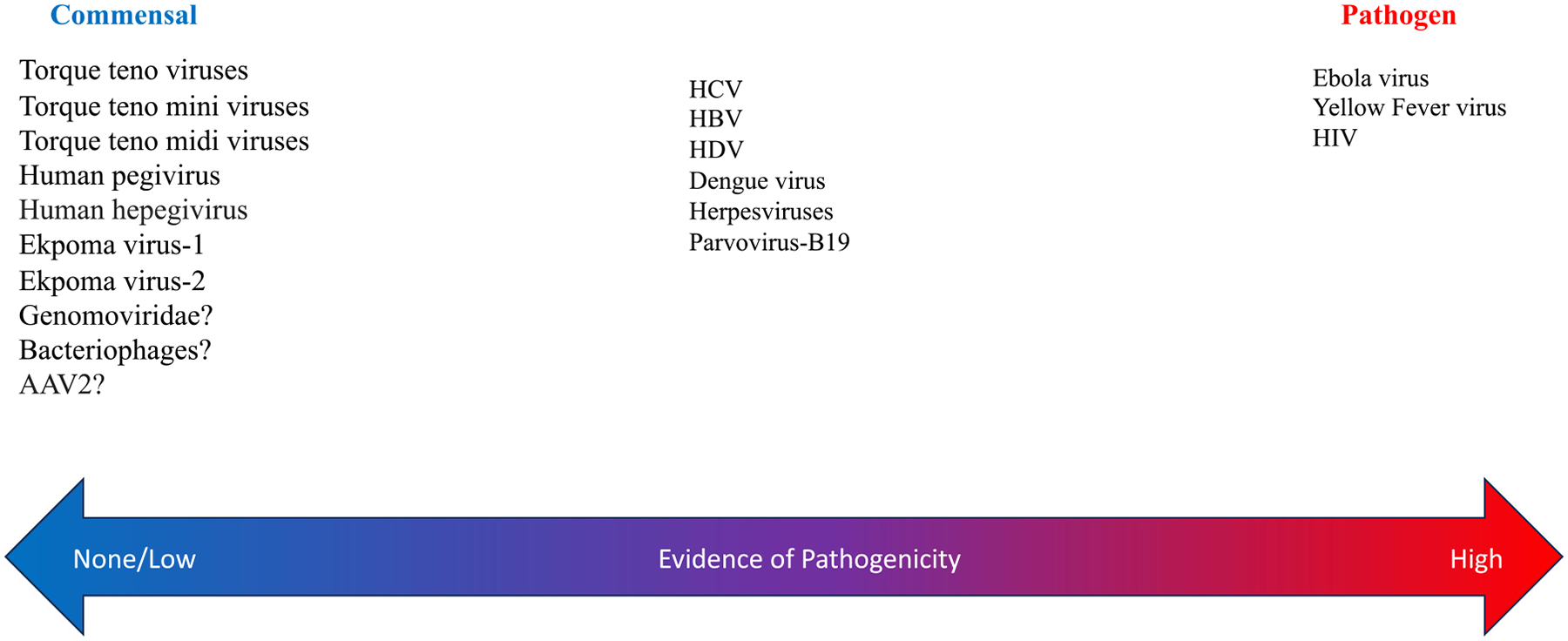
A conceptual framework for understanding the human blood virome. Detection in blood of some viruses (like Ebola virus RNA) is always interpreted as a pathogen while others (like torque teno viruses) are considered commensals. Still others like HCV RNA may be detected when causing a disease like cirrhosis or be coincidentally found in a person in whom there is no current medical significance recognized. Specific (and variable) host-virus interactions determine these outcomes, a principle explaining why a commensal may also be a pathogen. This framework suggests the confidence in the designation of a virus as a commensal should be commensurate with the quality and quantity of medical research (which is generally low) and allows for changes as new information unfolds. This is not an exhaustive list and contains a representative list of eukaryotic viruses. AAV: Adeno associated virus; HCV: Hepatitis C Virus; HBV: Hepatitis B Virus; HDV: Hepatitis D Virus; HIV: Human Immunodeficiency Virus

Our conceptual framework of the virome assumes a wide spectrum of interactions with the human host, with some overlap and considerable uncertainty (Fig. 1). Establishing a convincing causal relationship between a virus and disease requires satisfaction of strict principles like Koch’s postulate or the Bradford Hill criteria. [44] Conversely, the confidence we have in the *absence* of pathogenicity (and in which of these categories a virus belongs) is only as strong as the evidence, which in most cases is limited and changing. For example, adeno-associated viruses are assumed to be commensals, and on that basis, preferred for gene therapy. Yet, in some children expressing HLA-DRB1*04:01 allele, it appears adenoassociated viruses might cause severe, and even fatal, hepatitis. [45]

Accordingly causal inferences can be initially challenging with chronic viral infections. Even with viruses like HIV and HCV for which the disease consequences are now evident and widely accepted, there were initial delays establishing the medical impact. In this framework, we interpret the limited data on members of the blood virome, recognizing that persistent detection in blood at least implies infection of host cells and justification for special consideration of significance as compared, for example, for viruses and phages recovered from the mucosal or skin surfaces.

The initial discovery of TTV in individuals with post-transfusion hepatitis led to the assumption anelloviruses caused hepatitis. [46,47] However, accumulating evidence suggested their detection in that setting may have been coincidental. There was certainly no evidence anellovirus infection triggered profibrotic liver disease pathways like hepatitis B virus or hepatitis C virus.

One hypothesis is that without causing disease per se, anellovirus abundance might reveal or shape the underlying immune control of the host. The evidence comes from transplant settings where transplant recipients with higher anellovirus burden were more likely to acquire other opportunistic infections than recipients with lower burdens. This finding is interpreted to reflect lower net immunity. [48] Conversely, recipients with lower levels of anellovirus burden were more likely to experience organ rejection, which would correspond to higher net immunity (albeit against a tissue vs virus). [49]

While these clinical findings have been confirmed, they remain largely unexplained and challenge the classification of anelloviruses as true commensals. If anelloviruses ‘harmlessly’ replicate in circulating immunologically active cells, fewer target cells crudely would be expected to be associated with lower anellovirus burden and lower, not higher, net immunity. If on the other hand, high anellovirus abundance signaled lower “immunity,” then anelloviruses are controlled by immunity shared with other microbes and/or directly reduce that immunity. Anti-anellovirus immunity might explain persistence of some but not most anellovirus lineages, and discordance between the anellovirus lineage in a donor inoculum and a recipient anellome. [17] Anti-anellovirus immunity is also presumed to drive the high level of variability observed in the ORF-1. [17,43,50]

However, to date, there is little direct evidence of measurable anti-anellovirus immunity. There was no evidence of upregulated ISG transcription in tissues containing TTV transcripts. [42] A comprehensive profiling of 32,960 anellovirus peptides revealed absence of antibody response to 85% of the library with 8.65% in two or more subjects and delayed antibody appearance (>100 days) following infection. [51] Similar studies on cell mediated immunity are lacking. The lack of a measurable immune response runs counter to the hypothesis of immune evasion. Clearly much more work is needed to understand this highly prevalent member of the human virome.

## 4. RNA commensals in human blood virome

The most commonly observed commensal RNA viruses belong to genus *Pegivirus* in the *Flaviviridae* family (Table 1). Other well-known members of family *Flaviviridae* include human pathogens like HCV, dengue virus, and yellow fever virus. Pegiviruses vary in size between 50 and 100 nm and contain a positive sense RNA. The first human pegivirus species, HPgV, was discovered simultaneously by two independent groups and named GBV-C and hepatitis G virus (HGV). [9,11] Subsequent phylogenetic analysis revealed GBV-C and HGV to be minor variants of the same species. Similarly in 2015, a novel pegivirus was discovered in transfusion recipients using metagenomic sequencing. [7, 8] The virus was determined to share similarities to both HCV and HPgV and is now called HHPgV.

Rhabdoviruses have a negative-sense, single-stranded RNA of 10.8–16.1 kb. This large family is composed of at least 275 species with the well-recognized human pathogens known by their bullet shaped morphology in electron micrographs. [52] The established human pathogens in this group belong to genera *Lyssavirus* (e.g., rabies virus) and *Vesiculovirus* (e.g.,chandipura virus). [52] Recently mNGS identified two rhabdoviruses, ekpoma virus 1(EKV-1) and 2 (EKV-2), belonging to genus *Tibrovirus* in the plasma of two healthy women in Ekpoma, Nigeria. [16] An additional EKV-2 sequence with limited description was deposited in GenBank (accession#MF079256) in 2018. Phylogenetic analysis suggests possible relation between EKV-1 and Tibrogargan virus (TIBV) while EKV-2 formed another branch with Bas-Congo Virus (BASV). [16] BASV has been identified in the blood of three individuals with acute hemorrhagic fever in Democratic Republic of Congo. [53] However, no causal link between BASV and acute hemorrhagic fever has been shown.

### 4.1. Non-human mammalian analogs

Pegivirus infections have also been observed in non-human mammals including horses and swine. Theiler’s diseases associated virus (TDAV) was identified as the etiological agent of Theiler’s disease in horses using mNGS. [54] TDAV infection is transmitted by products sourced from infected equine blood causing acute hepatitis and mortality. [54,55] Another distinct pegivirus, equine pegivirus, has also been described in horses with elevated liver enzymes and 15–32% of otherwise healthy animals. [56] Likewise, porcine pegivirus RNA has been detected in the serum of 2.2–15.1% of healthy pigs. [57,58] Additional pegivirus species have also been observed in primates, bats, and rodents. [37,59] Except for TDAV, no disease has been associated with the other pegiviruses.

Asymptomatic tibrovirus infections have been also identified in non-human mammals, especially in cattle (and the midges that feed on cattle). [52] The immense diversity of this genus was highlighted by the recent identification of a novel tibrovirus, Ticpantry virus 4, in a healthy sanctuary housed chimpanzee in Central Africa. [37]

### 4.2. Epidemiology

HPgV is blood-borne virus that may also be transmitted via sexual exposure. [60] Hence, certain groups like individuals on hemodialysis, solid organ recipients, persons with hemophilia, and people who inject drugs are at a higher risk of acquisition. [61] Due to shared mode of transmission, co-infection with HIV and HCV is common. [61] A recent meta-analysis of 35,468 blood volunteers revealed a global HPgV viremia of 3.1% (95% CI,2.4–4.1) while seroprevalence rates range from 10% to 15%. [61,62] Since the majority of HPgV infections are self-limited, the prevalence of viremia at any one time underestimates the overall risk. This paradigm was clearly seen in a PWID cohort in whom persistent HPgV RNA was detected in 28%. However, all but 5% of those without RNA had already been infected as indicated by HPgV antibodies. [63] Thus, there was clear evidence of near universal infection, spontaneous clearance in many, and establishment of lasting protective immunity.

Data are not available on the prevalence of HHPgV virus in the general population. Among PWID, Coller et al. identified a prevalence of 11.2% for makers of active and resolved HHPgV infection among past or current HCV infected PWID compared to 1.9% in HCV negative PWID. [64] Likewise, HHPgV was reported in 10.9% of Baltimore PWID. [6] As with HPgV, at least some HHPgV infections persist and HHPgV specific antibodies are detectable. [6,65] However, while E2 antibodies produced following HPgV infection is associated with recovery, co-detection of HHPgV RNA and antibodies is observed frequently (92.86%). [65]

Following the initial discovery of EKV-1 and EKV-2, seroprevalences of 5% and 45%, respectively, were described in 457 persons living in Nigeria. [16] In contrast, prevalence of ~1% were described for both viruses in 137 individuals from in the Unites States. In the Nigerian study high prevalence study, the authors considered cross reactivity with other rhadoviruses leading to an overestimation of EKV-1 and 2 infections. [16] The mode of transmission of EKV-1 and 2 in humans is currently unknown. Among non-human mammals tibrovirus are vector borne diseases, and it is possible that transmission to humans might be incidental.

### 4.3. Microbiology

Like other members of family *Flaviviridae*, pegiviruses encode a single multifunctional polyprotein that is cleaved by host and viral proteases. [61] Compared to HCV, HPgV lacks a core protein, has fewer predicted E2 glycosylation sites, and has a type III instead of a type IV IRES at the 5’ end. [61] The lack of a core protein leads to the possibility of the involvement of cellular derived macrovesicles in cell entry and release. Supporting this inference are data showing the presence of HPgV RNA in serum microvesicles obtained from infected individuals and in vitro infection of PBMC using HPgV containing microvesicles. [66]

HPgV RNA has been detected in many tissues including spleen, kidney, liver, saliva, and bone marrow. The possible binding of serum microvesicle to the universally low-density lipoprotein receptors on human cells might be responsible for the broad tropism of the virus. In vitro limited HPgV replication can be sustained in lymphocytes. [67]

Even less is known about HHPgV, which has been detected both in liver and lymphocytes. [6,68] One interesting aspect of HHPgV biology is the consistently observed strong association with ongoing HCV infection. HHPgV RNA abundance has been observed to track with HCV and decrease following direct acting antivirals that lower HCV RNA to undetectable levels. [69,70] There are notable examples of HHPgV in the absence of HCV RNA indicating that if there is viral dependence, it is not strict/necessary. [6,7]

HPgV and HHPgV have less genetic diversity than many other RNA viruses suggesting that the genomes might be subject to unusual evolutionary constraints. [61,69] A longitudinal study on a quadruple infected (HIV, HCV, HPgV, and HHPgV) subject observed similar frequency of minor variants for all four viruses at two timepoints tested. [69] However, HHPgV minor variants at the first time point did not lead to any changes in amino acid sequences at the second time point. This indirect evidence suggests that HHPgV might have an error prone RNA-dependent RNA polymerase (RdRp) like other RNA viruses. However, in vitro studies have indicated HHPgV RdRp to have a higher fidelity than HCV RdRp. [71] Thus, that combination of RdRp along with constraints on evolutionary space might contribute to the low sequence diversity among HHPgV isolates.

It has been difficult to characterize EKV-1&2 infections in the absence of isolates and limited cases of viremia. Using pseudotyped viruses, a study using recombinant vesicular stomatitis Indiana virus expressing G protein of EKV-1&2 observed viral entry and replication in 60 highly characterized cancer lines (NCI-60 panel). [52] However, these results should be interpreted with caution as the cell tropism data was not reflective of authentic tibrovirus infections when using previously isolated tibroviruses.

### 4.4. Medical significance

There is no known medical consequence of pegivirus infections in humans. Several studies have not sustained the link between HPgV infections and acute hepatitis. [72,73] Others have variably linked HPgV to lymphoma, an association confirmed in a recent meta-analysis (and challenging its designation as a commensal). [74]

One interesting association was found between HPgV and improved outcomes among people living with HIV (before effective antiretroviral therapy). [14] Several groups have shown that HPgV viremia is associated with lower amounts of immune activation in persons living with HIV compared to aviremic individuals. [61] The E2 protein has been shown to reduce activation of tyrosine Src kinase during TCR induced signaling. [61] HPgV infection of astrocytes and microglia have also been shown to be associated with downregulation of IRF1 transcripts and PMP70, a peroxisome associated gene, in brain tissue. [75] Thus, the varied effects of HPgV on the immune system may contribute to control of HIV-1 and affect immune surveillance that typically prevents oncogenesis.

Information on the medical significance of EKV-1&2 virus infection is lacking. A follow up of the two epkoma virus infected individuals found both individuals to aviremic but seropositive for their respective EKV infection. [16] Further characterization of ekpoma infections have been limited due to the absence of other well documented cases in humans.

## 5. Challenges and open questions

Despite the advances in molecular virology, there remain significant challenges and large gaps in our understanding of the blood virome. There are methodological limitations. [6,76,77] In one study comparing mNGS to targeted qPCR, reads for HIV and HCV were only reliably detected when RNA abundance was >4 log_10_ copies/ml. [6] Moreover, methods used for extraction and sequencing of viruses may impede detection of less abundant viruses or larger phages. [5]

Interpretation of mNGS results should also consider possibility of nucleic acid contamination that can be introduced during different steps. [78] While negative controls can account for some, the most definitive approach is to confirm findings in another blood specimen from the same person processed independently. While repeated detection of the same sequence using this approach can improve confidence, it can be impractical. Moreover, failing to detect the same sequence might be due to natural fluctuations in viremia, not contamination of the original. Single instances of detection of viremia that cannot be confirmed with repeated blood sampling can make it difficult to differentiate between replication in the human host (and is a member of the blood virome) or transient viremia after sporadic inoculation/ingestion. An example is the detection of insect- and bat-associated viruses in the blood of two healthy individuals. [79] Phylogenetic analysis pointed to consumption of food or water contaminated with excrement from infected mammals as a route of acquisition. [79] However, the high level of reads and presence of minor variants suggested replication in human tissues.

Since an individual’s virome exists in health, detection of a virus in association with disease can be mistakenly interpreted as causal, as illustrated by the example of HGV, later renamed HPgV. [11] Further, the expectation that most viruses that can cause disease will do so only in a subset of hosts underscores the challenge of “proving the negative”, that is, that these are commensals. Indeed, following cloning of HCV, the pathogenicity was debated due to delay in disease manifestation and lack of universal occurrence. The previously mentioned example of adeno-associated virus causing severe liver disease in a subset of children underscores how difficult it may be to conclude a virus is a commensal. [45]

It is also possible the commensal viruses can affect human physiology different from the infected human cell. E.g., the presence of insulin/IGF1-like peptides in viruses that functioned like ‘viral hormones.’ [80]

To overcome these limitations future studies can make more extensive use of negative controls and repeated sampling of human hosts. Studies can also explore the biological plausibility of putative findings. Rare disease linkages are likely to require nonconcurrent cohort (nested case control) approaches in which viromic analysis is performed on blood samples collected years (or decades) before a disease occurred and controls who never experienced the disease and be interpreted in light of comprehensive human genomic analyses. These are among the ‘next generation’ of studies that will be required to elucidate the significance of the blood virome.

## 6. Conclusion

The blood virome is a fascinating new frontier of the microbiome that is now opened by metagenomic sequencing but remains largely unexplored. Future research is needed to characterize all members of the human blood virome to understand their epidemiology, microbiology, their tri-kingdom (host-microbiome-virome) interactions, and medical/physiological consequences.

## Supplementary Material

1

## Figures and Tables

**Table 1:** Commensal blood virome sequences frequently identified in healthy humans.

Realm	Family	Genus	Genome	Species	Virus name(s)
*NA*	*Anelloviridae*	*Alphatorqueviruses*	ss circular DNA (−)	*Alphatorquevirus homin*	Torque teno virus
*NA*	*Anelloviridae*	*Betatorqueviruses*	ss circular DNA (−)	*Betatorquevirus homini*	Torque teno mini virus
*NA*	*Anelloviridae*	*Gammatorqueviruses*	ss circular DNA (−)	*Gammatorquevirus homidi*	Torque teno midi virus
*Monodnaviria*	*Genomoviridae*	*Gemykibivirus*	ss circular DNA (+/−)	*Gemykibivirus humas*	-
*Monodnaviria*	*Genomoviridae*	*Gemyvongvirus*	ss circular DNA (+/−)	*Gemyvongvirus humas1*	-
*Riboviria*	*Flaviviridae*	*Pegivirus*	ss linear RNA (+)	*Pegivirus hominis*	GBV-C, human pegivirus, pegivirus-C
*Riboviria*	*Flaviviridae*	*Pegivirus*	ss linear RNA (+)	*Pegivirus columbiaense*	human hepegivirus, human pegivirus 2, pegivirus-H
*Riboviria*	*Rhabdoviridae*	*Tibrovirus*	ss linear RNA (−)	*Tibrovirus alphaekpoma*	Ekpoma virus 1
*Riboviria*	*Rhabdoviridae*	*Tibrovirus*	ss linear RNA (−)	*Tibrovirus betaekpoma*	Ekpoma virus 2

NA: None assigned; ss: single stranded; −: negative-sense; +: positive-sense.
